# Cross‐Sectional Brain Age Assessments Are Limited in Predicting Future Brain Change

**DOI:** 10.1002/hbm.70203

**Published:** 2025-04-16

**Authors:** Max Korbmacher, Didac Vidal‐Pineiro, Meng‐Yun Wang, Dennis van der Meer, Thomas Wolfers, Hajer Nakua, Eli Eikefjord, Ole A. Andreassen, Lars T. Westlye, Ivan I. Maximov

**Affiliations:** ^1^ Department of Health and Functioning Western Norway University of Applied Sciences Bergen Norway; ^2^ Department of Neurology Neuro‐SysMed Center of Excellence for Clinical Research in Neurological Diseases, Haukeland University Hospital Bergen Norway; ^3^ Mohn Medical Imaging and Visualization Centre (MMIV) Bergen Norway; ^4^ Center for Lifespan Changes in Brain and Cognition, Department of Psychology University of Oslo Oslo Norway; ^5^ Max Planck Institute for Psycholinguistics Nijmegen the Netherlands; ^6^ Centre for Precision Psychiatry, Division of Mental Health and Addiction, Oslo University Hospital & Institute of Clinical Medicine University of Oslo Oslo Norway; ^7^ Department of Psychiatry and Psychotherapy, Tübingen Center for Mental Health University of Tübingen Tübingen Germany; ^8^ Columbia University Irving Medical Centre Columbia University New York City USA; ^9^ KG Jebsen Centre for Neurodevelopmental Disorders University of Oslo Oslo Norway; ^10^ Department of Psychology University of Oslo Oslo Norway

## Abstract

The concept of brain age (BA) describes an integrative imaging marker of brain health, often suggested to reflect aging processes. However, the degree to which cross‐sectional MRI features, including BA, reflect past, ongoing, and future brain changes across different tissue types from macro‐ to microstructure remains controversial. Here, we use multimodal imaging data of 39,325 UK Biobank participants, aged 44–82 years at baseline and 2,520 follow‐ups within 1.12–6.90 years to examine BA changes and their relationship to anatomical brain changes. We find insufficient evidence to conclude that BA reflects the rate of brain aging. However, modality‐specific differences in brain ages reflect the state of the brain, highlighting diffusion and multimodal MRI brain age as potentially useful cross‐sectional markers.

## Graphical abstract

Multimodal imaging data of 39,325 UK Biobank participants with 2,520 follow‐ups provide insufficient evidence to conclude that brain age reflects the rate of anatomical brain aging. However, modality‐specific differences in brain ages reflect the cross‐sectional state of the brain, highlighting diffusion and multimodal MRI brain age as potentially useful cross‐sectional markers.

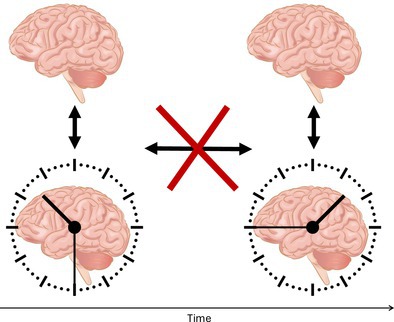

## 1 Introduction

Biomarkers which successfully characterize aging still need to be established. An emerging candidate for such a marker is the concept of biological brain age (BA). Algorithms that predict BA provide insight into the differences between imaging metrics of healthy populations and independent target populations, for example, presenting a certain pathology. BA can be predicted from different types of imaging data, such as different modalities or brain regions (Korbmacher et al. 2024a). The difference between BA and chronological age, called the brain age gap (BAG), has been used as a proxy for brain health. Previous studies identified the largest group‐level differences in BAG between healthy controls and individuals with neurodegenerative disorders (Franke and Gaser 2019; Kaufmann et al. 2019) which makes BAG particularly interesting in the context of aging; both healthy and pathological. Despite their cross‐sectional design, brain age studies often claim to examine aging processes. Conclusions about aging processes however require longitudinal study designs. As most brain age studies are cross‐sectional, the value of BAG in prognostics is unknown. To increase the clinical utility of BAG metrics, it is hence necessary to understand the degree to which cross‐sectional BAG can predict brain aging later in life. The need of a closer examination of the relationship between cross‐sectional BAG and longitudinal processes has also recently been highlighted by a lack of explanatory power of brain age of longitudinal processes when estimated based on $T_{1}$‐weighted magnetic resonance imaging (MRI) (Vidal‐Pineiro et al. 2021; Korbmacher et al. 2023a). Although radiomic features extracted from diffusion MRI and multimodal MRI have been shown to accurately predict age (Korbmacher et al. 2024a; Franke and Gaser 2019; De Lange et al. 2020), and potentially more accurately than $T_{1}$‐weighted MRI‐derived features (Korbmacher et al. 2024a), the value of such brain ages for longitudinal predictions has not been tested. Here, we capitalized on the largest accessible multimodal MRI dataset featuring $T_{1}$‐weighted and diffusion MRI from the UK Biobank including thousands of healthily aging participants. We tested the basic question to which extent BAs, trained on all available features of different MRI modalities or multimodal MRI, reflect longitudinal changes in brain age and brain features (i.e., brain aging). We attempted to focus on aging effects not influenced by specific pathologies or disorder to establish a baseline understanding of expectable aging effects and relationships to brain age. Our results present a weak correspondence between cross‐sectional brain age and longitudinal measures.

## 2 Results

**FIGURE 1:**
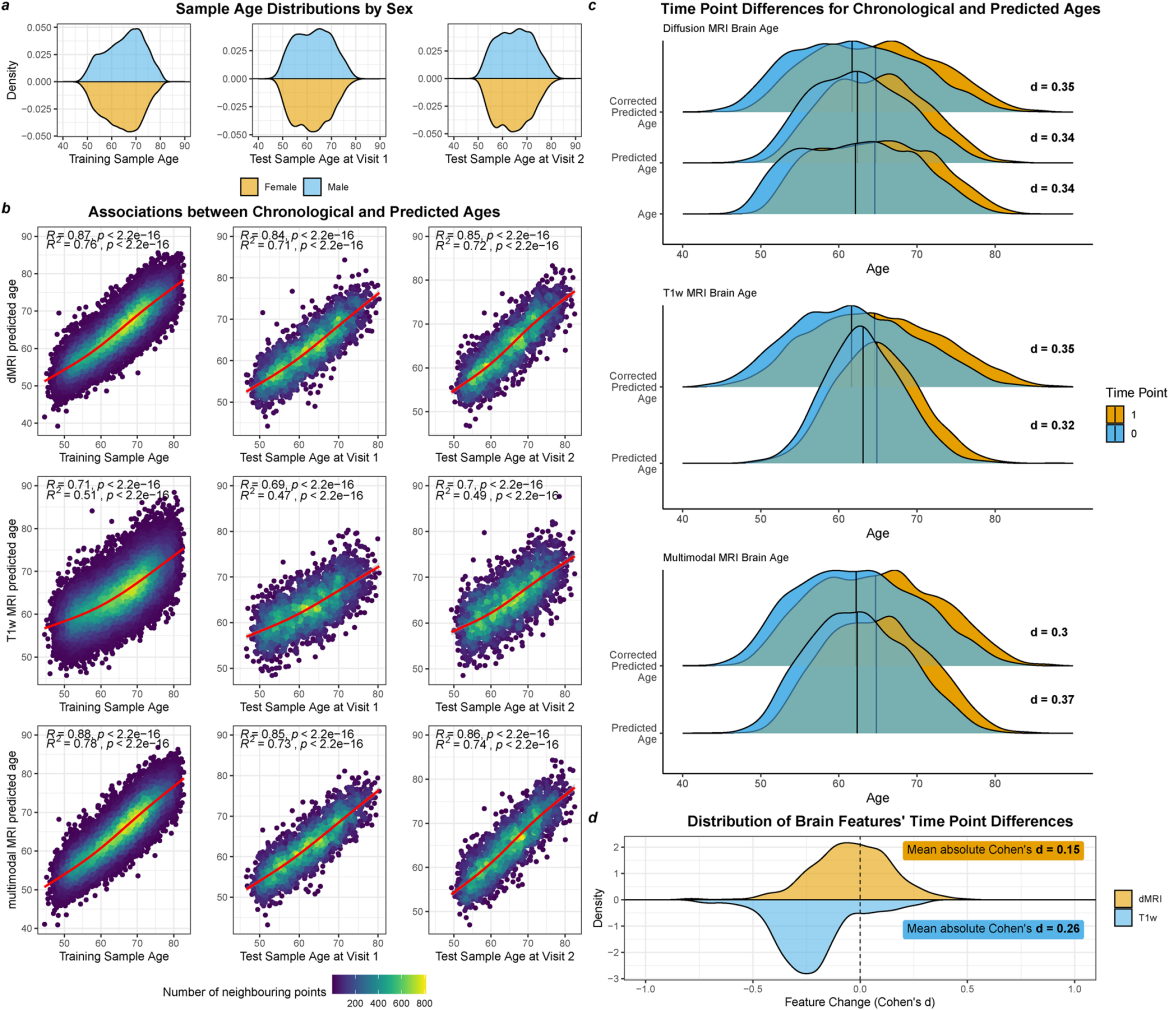
Training and test sample had similar characteristics and brain age was predicted with high accuracy in training and each test data time point individually. (a) Sample age distribution at each visit, separating the cross‐sectional training data from the longitudinal test data. (b) Model Performance for the training set, and the two test points for each MRI modality. Uncorrected estimates are presented, which were overlaid with a cubic spline with *k* = 4 knots. (c) Time point differences for age and both crude and age‐bias corrected BAs for each MRI modality indicated by Cohen's d (d). Distribution of effect sizes indicating the change in anatomical features of diffusion MRI (dMRI) and $T_{1}$‐weighted MRI (*T*_1w_). For additional associations between centercepts (i.e., averages between time points) and rate‐of‐change values see Table S6.

Despite the short inter‐scan interval (ISI), we could observe tissue maturation indicated by significant time‐point differences in MRI‐derived regional brain features (cortical thickness, surface area, cortical volume and diffusion metrics across brain‐regions, Figure 1d). Paired‐samples *t*‐test indicated that more than 90% of the $T_{1}$‐weighted, and more than 78% of the diffusion‐derived features changed significantly between time points ($\bar{|}d_{T_{1}w}|\;=0.26$, $\bar{|}d_{\text{dMRI}}|\;=0.15$; Figure 1d), with larger magnitude of these changes observed for $T_{1}$‐weighted (∣d∣=0.20−0.30; Figure S5) compared to diffusion metrics (∣d∣=0.12−0.16; see Figure S6 metric‐level changes). The features that showed significant change (*p* < 0.05, $N_{T_{1}w}$ = 202, $N_{\text{dMRI}}$ = 1618) between baseline and follow‐up were then used to compute principal components of the averages ($\bar{P}C$) and the annual rate of change of these features (ΔPC; Figures S1 and S3).

Figure 1 demonstrates that BA trained on cross‐sectional data can be applied in longitudinal data. Training and test sample characteristics were similar (Figure 1a, Table S1). BA predictions in the test sample were strongly associated with chronological age ($r_{\text{uncorrectedBA}}>0.47$, $r_{\text{correctedBA}}>0.78$; Figure 1b, Table S2) and correlated between time points (r>0.91; Table S2). The resulting BAGs were also strongly correlated between time points (r>0.80), reflecting the feature correlations between time points (${\bar{r}}_{T_{1}w}>0.85,{\bar{r}}_{\text{dMRI}}>0.76$), and BAGs increased significantly over time ($\beta_{\mathrm{std}}>0.32$ years, $p<2\times 10^{-10}$; Figure 1c, Table S3), indicating accelerated brain aging.

**FIGURE 2:**
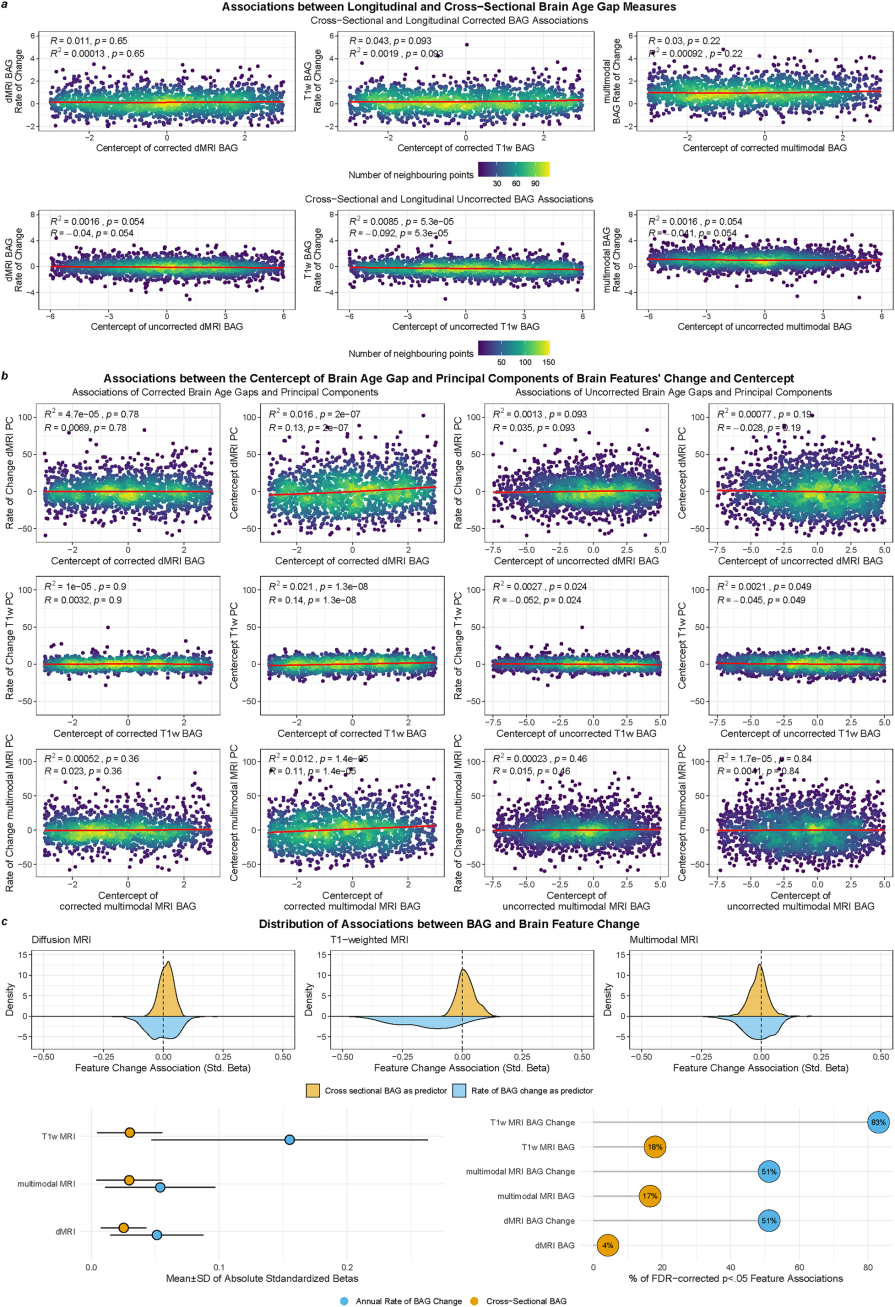
BAG is overall limited in reflecting brain change, yet, $T_{1}$‐weighted brain age reflects the strongest regional brain changes. (a) Associations between uncorrected $\bar{\mathrm{BAG}}$ and ΔBAG in the top row, and corrected associations in the bottom row. Associations were obtained specific to each modality: $T_{1}$‐weighted (*T*_1w_), diffusion (dMRI), and multimodal MRI. The displayed line fits were cubic splines with *k* = 4 knots. (b) Associations between the averages (proxy for cross‐sectional BA measures) of each modality‐specific BAG and PCs of both the averages and the annual rate of change in brain features. The left two columns present associations of uncorrected BAG estimates, and the right two columns of training‐sample age‐corrected BAG estimates, respectively. The displayed line fits were cubic splines with *k* = 4 knots. (c) Top row: Distribution of associations between corrected BAG and brain features and annual change of brain features (including associations with $p_{\mathrm{FDA}}<0.05$). Bottom left: Absolute mean and standard deviation of the associations between average and rate of change in corrected BAG and annual change of brain features. Bottom right: Percentage of significant associations between average and rate of change in corrected BAG and the annual change of brain features after Bonferroni‐correction.

Figure 2 illustrates that baseline (cross‐sectional) BAG, represented by the average of the BAGs ($\bar{\mathrm{BAG}}$), is limited in predicting longitudinal brain changes. For illustration, Figure 2 presents crude correlations—none of which were significant and positive when considering relationships between cross‐sectional and longitudinal measures. Focussing on the planned analyzes, using adjusted associations, within modalities, only the $T_{1}$‐weighted based corrected $\bar{\mathrm{BAG}}$ was significantly and positively associated with the annual rate of BAG change (ΔBAG; $\beta_{\mathrm{std}}=0.028\pm 0.148$; Figure 2a) and the principal component of longitudinal feature changes ΔPC ($\beta_{\mathrm{std}}=0.054\pm 0.015$; Figure 2b, Table S4a).

Yet, these relationships were not significant when controlling for interaction of ISI and $\bar{\mathrm{BAG}}$ (Table S4b). Only $T_{1}$‐weighted $\bar{\mathrm{BAG}}$ was associated with its principal component of change ΔPC ($\beta_{\mathrm{std}}=0.077\pm 0.022$). Cross‐sectional principal components ($\bar{P}C$) were limited in predicting principal components of longitudinal feature changes ΔPC (all associations were negative: $|\beta_{\mathrm{std}}|<0.063$; Table S4a,b). Finally, Figure 2 highlights the importance of age‐bias correction: corrected $\bar{B}\mathrm{AGs}$ were stronger related to ΔPCs and ΔBAGs than uncorrected $\bar{B}\mathrm{AGs}$. The associations between (longitudinal and cross‐sectional) measures were similarly weak across modalities (Figure 2b). Yet, the change in a larger number of $T_{1}$‐weighted features was significantly related to both $T_{1}$‐weighted ΔBAG (83%) and $\bar{\mathrm{BAG}}$ (18%; Figure 2c).

As a higher BAG can be expected at higher ages and potentially also the rate of change in BAG to accelerate, we show that our analyzes are independent of both, by correcting for the age bias (see Materials and methods), and by showing that $\bar{\mathrm{BAG}}$ can predict future changes in BAG, independent of ISI, between baseline and follow‐up. This was indicated by the effect of the interaction between the ISI and $\bar{\mathrm{BAG}}$ on ΔBAG being non‐significant (p>0.05; Table S5, Figure S2) when using either a linear or cubic interaction term, with the exception of uncorrected dMRI BAG (p=0.028). This indicates that the observed associations between cross‐sectional $\bar{\mathrm{BAG}}$ and ΔBAG were independent of the ISI in the current study, and hence not just an artifact of study design, age or aging.

BAG was limited in reflecting sub‐clinical health characteristics. Our sample was selected to not contain neurological or psychiatric disorders and showed relatively stable health based on various health indicators. These health indicators were limited in reflecting BAG. Health characteristics were evaluated by examining different risk factors for age‐related diseases and mortality, including cardiometabolics, depression, neuroticism, and polygenic risk scores (PGRS) of different disorders. Small associations were found between both BAGs and ΔBAG and different cross‐sectional health indicators (Figure S4a,b), including PGRS of common psychiatric disorders and Alzheimer's disease ($|\beta_{\mathrm{std}}|<0.06$, $p_{\text{Bonferroni}}>0.05$), clinically relevant state (depression rating) and trait (neuroticism) assessment scores ($\beta_{\mathrm{std}}<0.08$, $p_{\text{Bonferroni}}>0.05$), with larger group‐level differences for cardiometabolic factors hypertension and diabetes ($\beta_{\mathrm{std}}<0.60$, $p_{\text{Bonferroni}}<0.047$). Among the longitudinally available phenotypes, only waist‐to‐hip ratio (WHR), previously shown to be related to BAG (Korbmacher et al. 2023b), changed significantly between time points at the group level ($t=10.36,d=0.15,p<2.2\times 10^{-16},p_{\text{Bonferroni}}<2.2\times 10^{-16}$). However, while WHR showed a small, significant association with ${\mathrm{BAG}}_{T_{1}w}$ at baseline ($\beta_{\mathrm{std}}<0.10$), WHR changes were not predicted by BAGs or PCs (p>0.05; Figure S4a,b). Neuroticism ($t=2.83,d=0.04,p=0.005,p_{\text{Bonferroni}}=0.030$) and depression ($t=2.13,d=0.04,p=0.033,p_{\text{Bonferroni}}=0.198$) scores decreased over time, however, changes in these scores were also not found to be predicted by BAGs or PCs of brain feature change or averages (p>0.05).

## 3 Discussion

Taken together, our findings indicate that BAG is limited in reflecting longitudinal brain changes. Overall, (a) cross‐sectional BAGs presented small associations with longitudinal brain ages across modalities, (b) only BAGs from $T_{1}$‐weighted MRI features showed significant *but small* positive association with the respective longitudinal principal components, and (c) BAGs explained less than 1% of the variance of the mentioned longitudinal principal components and BAG change.

Yet, dMRI‐based cross‐sectional BAG correlated significantly with the annual change in around 38% of the region‐level features (at a relatively small average effect of $|\bar{\beta }|=0.140$). Assessing the rate of BAG change, $T_{1}$‐weighted BAG changes correlated significantly with the largest portion of regional brain change (83%), indicating that brain age reflects the state of the brain. Moreover, the cross‐sectional $T_{1}$‐weighted BAG associated with the largest proportion of change in brain features (18%). Hence, despite BAG correlating weakly with future change in BAG and future change principal components, a single‐time‐point $T_{1}$‐weighted BAG might allow to capture a portion of future changes in brain morphometry on the region level, whereas dMRI BAG is more reflective of the brain state. Future investigations might focus on constructing explainable brain age models which leverage region‐level data, and further investigate the potential of brain age in datasets with multiple follow‐ups. Alternatively, other markers which reflect a person's deviation from a norm defined by the characteristics of a training dataset, might be of interest.

Brain age allows reducing large amounts of information into a single personalized health score. Although brain age can be vulnerable to individual differences (Korbmacher et al. 2023a), such a single number is intuitive when set in contrast to a person's chronological age and does not require expert knowledge to be interpreted: a very high brain age in contrast to the chronological age might be alarming. Hence, brain age predictions hold the promise to provide additional information on routine clinical scans and, for example, support incidental findings. Closer examinations of how BAG reflects developmental trajectories under different conditions and on different samples offer opportunities for future research.

The identified increase in BAG over time indicates an acceleration of brain aging at a higher age. Such accelerated brain aging processes at older ages have also previously been highlighted in pathology‐free aging of white matter microstructure (Korbmacher et al. 2024b). In contrast, BAG during adulthood without pathology can be expected to be stable, since tissue changes remain small (Korbmacher et al. 2023a).

In the current study, examining adults from midlife to late life at two time points, we show significant brain changes as well as significant changes in BAG over time. Hence, BAG provides an indicator of the brain's morphometric state. Such a state has been shown to be influential for the future development of disorders, as indicated by disability accumulation in multiple sclerosis (Brier et al. 2023), the long‐term cognitive impact of stroke (Aamodt et al. 2023), or changes in dementia ratings (Tseng et al. 2022). Despite, for example, one study showing an increase in BAG over time in multiple sclerosis (Høgestøl et al. 2019), the observed increase in our non‐pathological sample casts doubt on the interpretation of the increase in the gap. At the same time, a study analyzing patient data presented that a faster brain age increase after stroke is linked to cognitive decline (Aamodt et al. 2023). The predictive utility of brain age in clinical samples may differ from that observed in the sample presented in this study, particularly when considering brain changes associated with specific disorders that a model might interpret as age‐related. This underscores the importance of model configuration, which is inherently dependent on the training data, including input features, processing pipelines, and potential sample biases. While it is possible that certain brain age modeling configurations could accurately reflect specific aging processes, current brain age estimations have yet to achieve this level of precision. Furthermore, the biological mechanisms underlying both increasing gaps in brain age predictions and the significance of baseline gaps for future developmental trajectories remain poorly understood and require further investigation.

We observed considerable modality‐dependent differences in brain ages. ${\bar{\mathrm{BAG}}}_{T1w}$ was most predictive of BAG change. Modality‐dependent differences might originate from the attempt to reduce a more complex feature space into single scores, such as brain age or principal components. This procedure might be a general limitation of the current brain age approaches, which usually use all available features, potentially losing information about independent groups of brain features. Utilizing approaches which maximize variability might be more appropriate (Smith et al. 2025). Moreover, the reliability of the longitudinal ΔBAG is unknown. Increasing the number of sampled time points for each participant might help to better characterize individual trajectories, particularly keeping measurement noise in mind which limits the possibility to detect subtle individual differences in developmental trajectories (Parsons and McCormick 2024). At the same time, null effects for $T_{1}$‐weighted BAG were previously presented in data with more observations and longer follow‐up times, questioning the utility of further exploration of approaches using all available brain features for dMRI and multimodal BAGs. On the other hand, the chosen cross‐sectional FreeSurfer‐based data processing pipeline for the $T_{1}$‐weighted MRI derived phenotypes introduces noisy estimates, as presented previously when comparing cross‐sectional and longitudinal FreeSurfer processing pipelines, with longitudinal estimates being more stable (Vidal‐Piñeiro et al. 2024; Wang et al. 2024). An introduction of such noise exacerbates the estimation of individual differences in brain change over the examined short time period. Hence, the extent of the presented brain changes, captured by $T_{1}$‐weighted MRI, remains unclear and needs to be interpreted with care, just as the presented annual rate of $T_{1}$‐weighted BAG change. Future modeling might focus on different spatial scales, such as voxel‐level analysis, and simultaneously on different biophysical modeling approaches to extract meaningful brain metrics. Moreover, as data selection and processing choices influence both brain age estimation cross‐sectionally (Korbmacher et al. 2024a, Korbmacher et al. 2024c), as well as longitudinal estimates of brain change (Vidal‐Piñeiro et al. 2024; Wang et al. 2024), more stringent tests are warranted varying different parameters systematically. A recent study (Smith et al. 2025) suggest that the feature selection and processing is of particular importance when attempting to relate brain age with longitudinal processes. This suggests that approaches not using all available brain (or other available) features, but rather a selection of variables representing different aging phenomena might be more successful in depicting longitudinal processes. This might in turn lead to practical applicability of brain age, when target phenomena are known. For example, if examining diabetes is the goal, a brain age from brain features sensitive to cardiometabolics, such as different white matter features (Korbmacher et al. 2024a, Korbmacher et al. 2025) might be useful to predict future brain ages or anatomical states. However, these findings need further empirical evaluation. Future modeling might also be served by either using more recent software versions, for example more recent than the legacy FreeSurfer version 5.3. Combining datasets processed with different FreeSurfer versions can also aid to increase cross‐sectional model performance (Korbmacher et al. 2024c). Similarly, additional explorations could focus on FreeSurfer analysis pipelines, for example, comparing longitudinal and cross‐sectional processing streams. Open questions on how FreeSurfer version differences and processing pipelines in general influence the relationship between cross‐sectional and longitudinal brain age measures provide opportunities for further explorations.

While our sample provides relatively large statistical power, the generalisability of our findings is still limited by a general positive health bias in the UK Biobank. Due to attrition effects, survival bias, and other common effects of longitudinal sampling, the positive health bias might be even stronger in the examined longitudinal portion of the UK Biobank. We examined various brain changes indicating brain aging processes. However, it cannot be excluded that these changes are differently pronounced in more representative samples or specific patient samples. As a consequence, this leaves also open whether BAG associates differently with changes in the presented health characteristics when using other similar samples. There is hence room for numerous potential follow‐up studies utilizing longitudinal data to explore BAG or other brain‐based predictive models and manipulating various parameters.

Despite the outlined limitations in predicting brain change in this study, brain age holds the potential to be a useful clinical marker. Different cohort‐ and phenomenon‐specific configurations of brain age might still prove the marker's prognostic value by predicting future aging processes. Following our findings, which present weak links between cross‐sectional and longitudinal BAGs, the cross‐sectional BAG still contains useful and simple‐to‐understand information about a patient's brain health. Such information could be automatically computed at the scanner and serve as Supporting Information when assessing the scan. However, the score lacks specificity. Hence, for clinically meaningful conclusions either brain age needs to be evaluated in the context of other variables, or conceptualized differently.

In conclusion, we find that cross‐sectional BAG estimates, currently commonly estimated from all available brain features, are limited in reflecting future brain changes. This limits the potential of such BAG for longitudinal inference and establishing BAG as a biomarker. During generally pathology‐free aging, BAG is not stable but increases, together with morphometric changes, potentially due to accelerated aging. Yet, only dMRI‐based BAG also reflected regional morphometric changes. These findings provide new and more pronounced insights into the mechanism of BAG. For example, a higher BAG does not automatically indicate the presence of a disorder, which would, however, be crucial for diagnostics. Instead, the observed modality dependencies suggest that dMRI and multimodal BAGs reflect the morphometric state, which is influenced by early life factors (Vidal‐Pineiro et al. 2021). DMRI BAG might reflect regional brain changes better than the other approaches. This more nuanced understanding of BAG underscores the need for closer examinations of the biological underpinnings of BAG to aid the general interpretation of the marker and to increase clarity around BAG's clinical utility.

## 4 Materials and Methods

### 4.1 Sample Characteristics

We obtained UKB data (Alfaro‐Almagro et al. 2018) containing dMRI data of N = 46,637 cross‐sectional datasets, of which N = 4,871 entailed data available at two time points, and N = 48,044 $T_{1}$‐weighted MRI datasets of which N = 4, (ref 11/NW/0382) 960 were followed up. Participant data were excluded when consent had been withdrawn, or data quality deemed to be insufficient based on the YTTRIUM method (Maximov et al. 2021) applied to dMRI data, and for $T_{1}$‐weighted data based on Euler numbers (Rosen et al. 2018), leading to exclusions when three standard deviations from the mean were exceeded. Additionally, we excluded participants which were diagnosed with any mental and behavioral disorder (ICD‐10 category F), disease of the nervous system (ICD‐10 category G), and disease of the circulatory system (ICD‐10 category I) from the training sample. The remaining datasets, after the exclusions were applied, entailed N = 36,805 purely cross‐sectional participants (52.17% females), which were used as a training set. The participants in the training sample were on average 64.79±7.70 years old (range: 44.57−82.75 years), with MRI scans obtained at four sites: (1) Cheadle (57.81%), (2) Newcastle (25.34%), (3) Reading (16.70%), and Bristol (0.15%). The independent testing set, not being a subset of the above mentioned training data, consisted of N = 2,520 participants (52.17% females) aged 62.26±7.19 years at baseline (range: 46.12−80.30 years), and at time‐point two, the mean age was 64.67±7.11 years (range: 49.33−82.59 years), indicating an average age difference of ΔAge=2.45±0.75 years (range: 1.12−6.90 years). The test data were collected at three sites: (1) in Cheadle (57.36%), (2) Newcastle (37.04%), and (3) Reading (5.60%).

### 4.2 MRI Acquisition and Post‐Processing

UKB MRI data acquisition procedures and protocols are described elsewhere (Alfaro‐Almagro et al. 2018; Miller et al. 2016; Sudlow et al. 2015) (https://www.fmrib.ox.ac.uk/ukbiobank/protocol/.) Briefly, the diffusion protocol consisted of two b‐values (1000 and 2000 s/mm (Franke and Gaser 2019)) with 50 non‐coplanar diffusion weighting gradients per shell. After access to the raw dMRI data was obtained, we processed the data using an optimized pipeline (Maximov et al. 2019). The pipeline includes corrections for noise (Veraart et al. 2016), Gibbs ringing (Kellner et al. 2016), susceptibility‐induced and motion distortions, and eddy current artifacts (Andersson and Sotiropoulos 2016). Isotropic 1 mm^3^ Gaussian smoothing was carried out using FSL's (Smith et al. 2004; Jenkinson et al. 2012) *fslmaths*. Employing the multi‐shell data, Diffusion Tensor Imaging (DTI), Diffusion Kurtosis Imaging (DKI) (Jensen et al. 2005) and White Matter Tract Integrity (WMTI) (Fieremans et al. 2011) metrics were estimated using Matlab 2017b code (https://gitgub.com/NYU‐DiffusionMRI/DESIGNER). Spherical mean technique SMT (Kaden et al. 2016a), and multi‐compartment spherical mean technique (mcSMT) (Kaden et al. 2016b) metrics were estimated using original code (https://github.com/ekaden/smt) (Kaden et al. 2016a, 2016b). Estimates from the Bayesian Rotational Invariant Approach (BRIA) were evaluated by the original Matlab code (https://bitbucket.org/reisert/baydiff/src/master/) (Reisert et al. 2017).

$T_{1}$‐weighted images were obtained at 3 T on a Siemens Skyra 3 T running VD13A SP4 (as of October 2015), with a standard Siemens 32‐channel radio frequency receive head coil using a 3D MPRAGE sequence (sagittal, in‐plane acceleration iPAT = 2, prescan‐normalize) at 1 × 1 × 1 mm^3^, field‐of‐view: 208 × 256 × 256 matrix, taking approximately 5 min. The $T_{1}$‐weighted images were processed using the cross‐sectional FreeSurfer (version 5.3) (Fischl 2012) automatic *recon‐all* pipeline for a cortical reconstruction and subcortical segmentation of the $T_{1}$‐weighted images (http://surfer.nmr.mgh.harvard.edu/fswiki) (Dale et al. 1999).

In total, we obtained 26 WM metrics from six diffusion approaches (DTI, DKI, WMTI, SMT, mcSMT, BRIA; see for overview Table S8). In order to normalize all metrics, we used Tract‐based Spatial Statistics (TBSS) (Smith et al. 2006), as part of FSL (Smith et al. 2004; Jenkinson et al. 2012). In brief, initially all brain‐extracted (Smith 2002) fractional anisotropy (FA) images were aligned to MNI space using non‐linear transformation (FNIRT) (Jenkinson et al. 2012). Following, the mean FA image and related mean FA skeleton were derived. Each diffusion scalar map was projected onto the mean FA skeleton using TBSS. To provide a quantitative description of diffusion metrics at a region level, we used the John Hopkins University (JHU) atlas (Mori et al. 2006), and obtained 48 white matter regions of interest (ROIs) and 20 tract averages based on a probabilistic white matter atlas (JHU) (Hua et al. 2008) for each of the 26 metrics. Altogether, 1794 diffusion features were derived per individual [26 metrics × (48 ROIs + 20 tracts + 1 global skeleton mean value)]. For *T*_1_‐weighted data, we applied the Desikan‐Killiany Atlas (Desikan et al. 2006) to obtain regional estimates of thickness, area, and volume, leading to 208 features [3 metrics × (34 ROIs + 2 global mean values (left and right hemispheres))]. This results in a total of 2002 multimodal MRI features per individual.

TBSS has previously been validated as cross‐sectionally and longitudinally reliable under various different conditions such as different age groups or scanner settings (Madhyastha et al. 2014; Merisaari et al. 2019; Rosberg et al. 2022; Melzer et al. 2020). Similarly, FreeSurfer estimated $T_{1}$‐weighted features show high intra‐class correlations (Knussmann et al. 2022; Liem et al. 2015). Yet, it has been suggested that FreeSurfer's more recent longitudinal analysis stream provides more accurate cortical reconstructions (Vidal‐Piñeiro et al. 2024; Wang et al. 2024). For consistency between longitudinal and cros‐sectional sample processing, but also due to limited computational resources, we provide data from the cross‐sectional FreeSurfer stream only.

### 4.3 Cardiometabolic Risk Factors

We used a selection of cardiometabolic risk factors, which have associations with BAG and are relevant to brain aging (Korbmacher et al. 2024a; Mo et al. 2023; Jagust et al. 2005; Habes et al. 2023; Jochemsen et al. 2013). Smoking, hypertension, and diabetes were binary and the waist‐hip ratio (WHR) is a scalar value.

### 4.4 Depression and Neuroticism Scores

Depression scores were computed using the Recent Depressive Symptoms (RDS‐4) score (fields 2050, 2060, 2070, 2080), which was suggested in a previous investigation using UKB imaging data (Dutt et al. 2022). Neuroticism scores (UKB data‐field 20,127) were derived as a summary score from the Eysenck Neuroticism (N‐12) inventory, which includes items describing neuroticism traits.

### 4.5 Polygenic Risk Scores (PGRS)

We estimated PGRS for each participant with available genomic data, using PRSice2 (Choi and O'Reilly 2019) with default settings. As input for the PGRS, we used summary statistics from recent genome‐wide association studies of Autism Spectrum Disorder (ASD) (Autism Spectrum Disorders Working Group of The Psychiatric Genomics Consortium 2017), Major Depressive Disorder (MDD) (Wray et al. 2018), Schizophrenia (SCZ) (Trubetskoy et al. 2022), Attention Deficit Hyperactivity Disorder (adHD) (Demontis et al. 2019), Bipolar Disorder (BIP) (Mullins et al. 2021), Obsessive Compulsive Disorder (OCD) (Arnold et al. 2018), Anxiety Disorder (ANX) (Otowa et al. 2016), and Alzheimer's Disease (ad) (Wightman et al. 2021). We used a minor allele frequency of 0.05, as the threshold most commonly used in PGRS studies of psychiatric disorders.

While psychiatric disorders were *p*‐values thresholded at *α* = 0.05 (Autism Spectrum Disorders Working Group of The Psychiatric Genomics Consortium 2017; Wray et al. 2018; Trubetskoy et al. 2022; Demontis et al. 2019; Mullins et al. 2021; Arnold et al. 2018; Otowa et al. 2016; Wightman et al. 2021; Lambert et al. 2013), recommendations for AD ($\alpha =1.07^{-4}$) (Clark et al. 2022) lead to the application of a lower threshold of α=0.0001, with the goal of optimizing signal to noise in comparison to previously used α=0.001 (Euesden et al. 2015). The goal of the PGRS estimation was to relate the PGRS to cross‐sectional and longitudinal BAG and the principal components. PGRS data were available for N = 2166 of the longitudinal datasets (after exclusions).

### 4.6 Principal Components (PC) of Brain Physiology

We conducted six principal component analyzes, one for the averages of the features and one for the annual rate of change of features for each modality: (1) $T_{1}$‐weighted, (2) diffusion, and (3) multimodal MRI features (as described in the MRI acquisition and processing section). The first component of each of the PC analyzes was selected (rate of change components (ΔPC): $T_{1}$‐weighted *R* ^2^ = 18.5%, dMRI *R* ^2^ = 23.5%, multimodal *R* ^2^ = 20.8; average components ($\bar{P}C$): $T_{1}$‐weighted *R* ^2^ = 30.5%, dMRI *R* ^2^ = 39.1%, multimodal *R* ^2^ = 34.6; see Figure S1). For categorical feature contributions of the different approaches to the first two components per modality see Figure S3, and for the relative contribution by feature type see Figure S7. In brief, contributions of feature classes across regions (e.g., forceps fractional anisotropy) to PCs were relatively evenly distributed. This suggests that there is not a single dominantly contributing feature for dMRI and multimodal components. Yet, thickness contributed more than volume and surface area for the first $T_{1}$‐weighted MRI component (Figures S3, S7).

### 4.7 Statistical Analyzes

All statistical analyzes were carried out using R version (v4.2.0, www.r‐project.org), Python (v3.7.1) and FSL (v6.0.2) (Jenkinson et al. 2012). P‐values which adjusted for multiple comparison using FDR‐correction (Benjamini and Hochberg 1995) are marked with $p_{\mathrm{FDR}}$, and p‐values corrected using the Bonferroni method are marked with $p_{\text{Bonferroni}}$. Standardised regression coefficients are labeled as $\beta_{\mathrm{std}}$, unstandardized coefficients as β. For example, in a regression function $\hat{y}=\beta_{0}+\beta_{1}\times x+\epsilon$, $\beta_{1}$ is the slope, with $\beta_{0}$ the intercept, and ϵ the modeling error. When scaling variables to a standardized value (*M* = 0, SD = 1), $\beta_{\mathrm{std}}$ can be obtained. The proportion of true null effects/*p*‐values was estimated using the Storey–Tibshirani method (Storey and Tibshirani 2003). An overview of the analysis protocol is given in Note S1.

#### 4.7.1 Brain Age Prediction

Brain age predictions refer to age predictions from a trained model in unseen data based on a set of MRI features, here, regional brain metrics representing different gray and white matter microstructure characteristics. A higher predicted age indicates that the model assumes this person is older, based on the presented brain features.

As the goal is to train models which are generalisable, we estimated the power of our model to do so under varying assumptions of parameter shrinkage, which quantifies the extent to which the model is generalisable. A larger shrinkage indicates lower generalisability. With the conservative assumption of a large to extremely large parameter shrinkage, for example, of 30%, 40%, 50%, and 60%, we estimated required training sample sizes of 14,826, 19,522, 25,848, and 35,117 participants to train a BA model on the selected maximum of 2002 features (the multimodal MRI brain age model), respectively, using the *pmsampsize* package (Riley et al. 2019).

We tested several algorithms, including eXtreme Gradient Boosting (XGBoost) (Chen and Guestrin 2016), the least absolute shrinkage and selection operator (LASSO) (Tibshirani 1996), and simple linear regression models using k‐fold nested cross‐validation with included hyperparamenter tuning on five inner and ten outer folds. Across all algorithms tested, linear regression models performed best in terms of variance explained and correlations of brain age predictions and chronological age and commonly used error metrics, including Root Mean Squared Error (RMSE), and Mean Absolute Error (MAE) on the training sample and were, therefore, used to predict BA (Table S7). The superiority of linear models was also underscored by a lower parameter shrinkage when predicting in the test data. These predictions are presented in the main text, whereas the results from the other algorithms are presented in the Supplement. Altogether, 2002 features (i.e., brain regional metrics based on $T_{1}$‐weighted or dMRI measures) were used per individual. After the training procedure was completed on the participants for which only a baseline scan was available, we predicted BA in the remaining participants (N = 2678) with tow available data points for each of these two study time points (baseline and follow‐up).

We calculated corrected BA estimates by first calculating the intercept (α) and slope (β) of the linear associations between predicted BA ($\gamma_{\text{train}}$) and chronological age ($\Omega_{\text{train}}$) in the *training* (baseline) sample (Equation 1):

(1) $${\hat{\gamma }}_{\text{train}}=\alpha +\beta \times \Omega_{\text{train}}.$$

The calculated intercept (α) and slope (β) from the training sample were then used to estimate a corrected BAG (BAGc), as previously suggested (de Lange et al. 2022), from the predicted age ($\gamma_{\text{test}}$) and chronological age ($\Omega_{\text{test}}$) separately in each of data points of the *testing* (longitudinal) sample:

(2) $$\begin{array}{l} B\hat{A}\mathrm{Gc}=\gamma_{\text{test}}+\left(\Omega_{\text{test}}-\left(\alpha_{\text{training}}+\beta_{\text{training}}\times \Omega_{\text{test}}\right)\right)-\Omega_{\text{test}}. \end{array}$$

We present the results for both corrected and uncorrected BAG.

As a control, we randomly split the longitudinal data into equal parts, trained models and predicted in $N_{\mathrm{TP}1-2}$ = 1339 at each time point within the same individuals (due to the high dimensionality of the dMRI and multimodal data in contrast to the degrees of freedom, only $T_{1}$‐weighted data were considered; Tables S9 and S10). These predictions did not only show large variability but also contra‐indicative trends of decreasing brain age over time. Hence, these values were not considered for further analyzes.

#### 4.7.2 Rate of Change and Averages

In order to investigate how single time point BA predictions relate to longitudinal changes in BA and features, we estimated the annual rate of change and averages in both features and BAs. Averages were used to establish cross‐sectional proxies (of the BAG, PCs, and brain features) which are statistically independent from the annual rate of change. Averages are the average of two measures without considering the inter‐scan interval (ISI). The annual rate of change, on the other hand, is the difference of the BAG at each time point and has the ISI as denominator.

#### 4.7.3 Exploratory Analyzes

Time point correlations between brain ages at each time point were assessed using uncorrected Pearson's correlations. To assess time point (TP) difference in the (a) corrected and (b) uncorrected brain age gaps (BAG), we used mixed linear models (MLMs) with ID, Site, Age, Sex, and the Age*Sex interaction as fixed effects, the subject/*ID* as random effect (u), and the subject residuals (e).

(3) $$\begin{array}{c} BÂG=\beta_{0}+\beta_{1}\times \mathrm{TP}+\beta_{2}\times \mathrm{Age}+\beta_{3}\times \mathrm{Age}*\mathrm{Sex}\\ \;+\beta_{4}\times \mathrm{Sex}+\beta_{5}\times \text{Site}+u_{\mathrm{ID}}+e \end{array}$$

We used paired samples t‐tests to assess features changes over time. Changing features were included in the principal components analyzes.

We evaluated the association of BA average or the annual rate of change in BA with (a) the first cross‐sectional principal component (of the averages of features) and (b) the principal component of features' annual rate of change (PC), correcting for the ISI.

(4) $$\begin{array}{c} BÂG=\beta_{0}+\beta_{1}\times \mathrm{PC}+\beta_{2}\times \mathrm{ISI}+\beta_{3}\times \mathrm{Age}\\ \;+\beta_{4}\times \mathrm{Sex}+\beta_{5}\times \mathrm{Age}*\mathrm{Sex}+u_{\text{Site}}+e \end{array}$$

When assessing how (a) the average of the BAG and (b) the annual rate of change in BAG (BAG) reflect brain features and change in brain features (F), to ensure model convergence, we used simple linear models.

(5) $$\begin{array}{c} \hat{F}=\beta_{0}+\beta_{1}\times \mathrm{BAG}+\beta_{2}\times \mathrm{ISI}+\beta_{3}\times \mathrm{Age}\\ \;+\beta_{4}\times \mathrm{Sex}+\beta_{5}\times \mathrm{Age}*\mathrm{Sex}+\beta_{6}\times \text{Site} \end{array}$$

Finally, we explored the associations between PC, BAG, and their annual rates of change $\bar{\mathrm{BAG}},\Delta \mathrm{BAG},\bar{P}C,\Delta \mathrm{PC}$ (four outcome variables), and time‐point specific principal components ${\mathrm{PC}}_{\mathrm{TP}1}$ and ${\mathrm{PC}}_{\mathrm{TP}2}$ and BAGs ${\mathrm{BAG}}_{\mathrm{TP}1}$ and ${\mathrm{PC}}_{\mathrm{TP}2}$ (another four outcome variables; all summarized in the formula as PC/BAG) with pheno‐ and genotypes (P/G), including PGRS of psychiatric disorders and Alzheimer's, depression and neuroticism scores, and cardiometabolic risk factors.
(6)

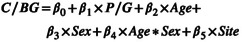




The associations between PGRS and rate of change of PC and BAG had the lowest statistical power due to the limited availability of participant's genetic data (N=2,160). We conducted a power analysis to ensure we would be able to detect meaningful effect sizes. We aimed for a power of 80%, and an α‐level of 0.05 in simple linear regression models, as described above, indicating that effect sizes as small as Cohen's $f^{2}=0.006$ can be detected, corresponding to a Pearson's correlation coefficient of r=0.006 or Cohen's d=0.012.

#### 4.7.4 Confirmatory Analyzes

We tested several preregistered hypotheses (see https://aspredicted.org/7bd4e.pdf)^1^. First, to test whether there were relationships between cross‐sectional and longitudinal measures of the obtained BAGs and PCs, we associated their averages (Wainer 2000) and annual rates of change. We ran first MLMs predicting the annual rate of BAG changes (${\mathrm{BAG}}_{\Delta }$) from cross sectional BAG ($B\hat{A}G$). We also predicted the longitudinal principal component of brain feature changes (${\mathrm{PC}}_{\Delta }$) from the cross‐sectional principal component ($\hat{P}C$) controlling for the inter‐scan interval (ISI), age, sex, and the age‐sex interaction as fixed effects, and scanning site as random effect (Equations (7) and (8)).
(7)

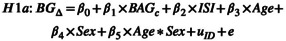



(8)

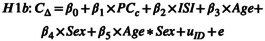




To test the interaction effect of ISI and the respective cross‐sectional measures (${\mathrm{BAG}}_{c}$ and ${\mathrm{PC}}_{c}$) we used generalized additive models (GAM) taking the same form of Equation (8), yet introducing a spline chaining k = 4 cubic functions s on the interaction of interest:
(9)

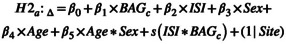



(10)

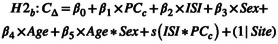




## Author Contributions

Max Korbmacher: study design, software, formal analysis, visualisations, project administration, writing – original draft, writing – review and editing. Didac Vidal‐Pineiro: study design, software, writing – review and editing. Meng‐Yun Wang: writing – review and editing. Dennis van der Meer: software, writing – review and editing. Thomas Wolfers: writing – review and editing. Hajer Nakua: writing – review and editing. Eli Eikefjord: writing – review and editing, funding acquisition. Ole A. Andreassen: writing – review and editing, funding acquisition. Lars T. Westlye: writing – review and editing, funding acquisition. Ivan I. Maximov: study design, data preprocessing and quality control, writing – review and editing, funding acquisition.

## Conflicts of Interest

Ole A. Andreassen has received a speaker's honorarium from Lundbeck and is a consultant to Coretechs.ai. The other authors declare no conflicts of interest.

## Supporting information


**Data S1.** Supporting Information.

**Data S2.** Supporting Information.

## Data Availability

This study has been conducted using UKB data under Application 27412. All raw data are available from the UKB (www.ukbiobank.ac.uk). UK Biobank has approval from the North West Multi‐centre Research Ethics Committee (MREC) as a Research Tissue Bank (RTB) approval. The raw and processed UK Biobank MRI data are protected and are not openly available due to data privacy laws. Unfortunately, the most recent changes in UKB policy urge us to delete all UKB data from our servers, limiting the computational reproducibility of our analyzes and future research based on our data. Updated procedures on how to obtain indirect data access against an access fee might be available on the UKB website (see https://www.ukbiobank.ac.uk/enable‐your‐research/apply‐for‐access).

## Endnotes

^1^
Note: Instead of estimating brain ages for dMRI only, we also used *T*_1_‐weighted and multimodal data as well. For more meaningful results, we did not conduct a genome‐wide association study with brain age and used instead polygenic risk scores of psychiatric disorders and Alzheimer's disease, as well as cardiometabolics and depression and neuroticism scores.
