# Supplementary material for: Cross‐Sectional Brain Age Assessments Are Limited in Predicting Future Brain Change
Source: Hum Brain Mapp. 2025 Apr 16;46(6):e70203. doi: 10.1002/hbm.70203 (PMC12000824; doi:10.1002/hbm.70203)
Supplement: Supplementary file 1 — Data S1. Supporting Information. [file HBM-46-e70203-s002.pdf]

SUPPLEMENTARY INFORMATION

Supplementary information to the article "Cross-sectional brain age assessments are limited in predicting future brain change", Korbmacher et al., 2024

Contents

SUPPLEMENTARY TABLES **3**

Supplementary Table 1. Model performance for corrected and uncorrected predictions . . . 3

Supplementary Table 2. Brain age gap correlations (crude) between time points 4

Supplementary Table 3. Time point differences in brain age gaps . . . 4

Supplementary Table 4. Associations between cross-section and longitudinal measures (Principal Components and BAGs) . . . 4

Supplementary Table 5. Associations between cross-section and longitudinal measures (Principal Components and BAGs) accounting for the interaction of the inter-scan interval and $B\bar{A}G$ . . . 5

Supplementary Table 6. Associations between Brain Age Gaps (longitudinal and cross-sectional) and Principal Components (longitudinal and cross-sectional) . . . 6

Supplementary Table 7. Model performance comparison . . . 8

Supplementary Table 8. White matter features by diffusion approaches . . . 9

Supplementary Table 9. Within time point predictions from T1w data . . . 9

Supplementary Table 10. Between time point predictions from T1w data (uncorrected BAG) . . . 11

Supplementary Table 10. Between time point predictions from T1w data (corrected BAG) . . . 12

SUPPLEMENTARY FIGURES **13**

Supplementary Figure 1. Scree Plots for the Principal Components Analyses 13

Supplementary Figure 2. Contour plots visualising the interaction effect of inter-scan interval and cross-sectional BAG on the annual rate of change of BAG . . . 13

Supplementary Figure 3. Relative contribution of features to the first two principal components for each modality . . . 14

Supplementary Figure 4. Associations between PCs, BAG, annual BAG and PC change, and pheno- and geno-types of health . . . 15

Supplementary Figure 5. Time point differences in features by T1-weighted metrics and diffusion approaches . . . 17

Supplementary Figure 6. Time point differences in features by processing approach . . . 18

Supplementary Figure 7. Relative contributions of feature types to principal components by modality 21
Supplementary Figure 8. dMRI centercept BAG associations with the rate of change in dMRI features 22

SUPPLEMENTARY NOTES **22**
Supplementary Note 1. Analysis protocol 22

References **24**

SUPPLEMENTARY TABLES

Supplementary Table 1. Model performance for corrected and uncorrected predictions

LM Predictions	R^2	MAE	RMSE	Correlation	lower 95% CI	upper 95% CI	Correction
dMRI training	0.761	2.995	3.765	0.872	0.870	0.875	No
dMRI test visit 1	0.709	3.107	3.924	0.842	0.830	0.853	No
dMRI test visit 2	0.722	2.976	3.754	0.850	0.838	0.860	No
T1w MRI training	0.510	4.338	5.393	0.714	0.709	0.719	No
T1w MRI test visit 1	0.472	4.300	5.356	0.687	0.666	0.707	No
T1w MRI test visit 2	0.494	4.032	5.073	0.703	0.683	0.722	No
multimodal MRI training	0.779	2.885	3.619	0.883	0.881	0.885	No
multimodal MRI test visit 1	0.729	3.012	3.773	0.854	0.843	0.864	No
multimodal MRI test visit 2	0.739	2.893	3.644	0.860	0.849	0.870	No
dMRI training	0.846	2.608	3.285	0.920	0.918	0.921	Yes
dMRI test visit 1	0.811	2.628	3.373	0.901	0.893	0.908	Yes
dMRI test visit 2	0.819	2.605	3.294	0.905	0.898	0.912	Yes
T1w MRI training	0.800	3.047	3.851	0.894	0.892	0.896	Yes
T1w MRI test visit 1	0.790	2.851	3.587	0.889	0.880	0.897	Yes
T1w MRI test visit 2	0.784	3.004	3.813	0.885	0.877	0.893	Yes
multimodal MRI training	0.853	2.538	3.195	0.924	0.922	0.925	Yes
multimodal MRI test visit 1	0.812	3.161	3.954	0.901	0.893	0.908	Yes
multimodal MRI test visit 2	0.824	2.597	3.272	0.908	0.901	0.914	Yes

R^2 = variance explained, MAE = Mean Absolute Error, RMSE = Root Mean Squared Error, 95% CI = 95% Confidence Interval.

Supplementary Table 2. Brain age gap correlations (crude) between time points

Modality	Variable	Correlation	Lower 95% CI	Upper 95% CI
dMRI	Predicted Age	0.926	0.920	0.932
dMRI	Corrected Predicted Age	0.950	0.946	0.954
dMRI	BAG	0.814	0.800	0.827
dMRI	Corrected BAG	0.755	0.738	0.772
T1w MRI	Predicted Age	0.912	0.905	0.918
T1w MRI	Corrected Predicted Age	0.960	0.957	0.963
T1w MRI	BAG	0.909	0.902	0.916
T1w MRI	Corrected BAG	0.829	0.816	0.841
multimodal MRI	Predicted Age	0.934	0.929	0.939
multimodal MRI	Corrected Predicted Age	0.956	0.952	0.959
multimodal MRI	BAG	0.807	0.793	0.820
multimodal MRI	Corrected BAG	0.756	0.739	0.772

95% CI = 95% Confidence Interval.

Supplementary Table 3. Time point differences in brain age gaps

	Beta	SE	p	R^2m	R^2c	Mean TP1	Mean TP2
dMRI Uncorrected	0.324	0.051	2.20×10^{-10}	0.250	0.815	0.419	0.093
dMRI Corrected	0.324	0.051	2.20×10^{-10}	0.009	0.756	-0.194	0.064
T1w Uncorrected	0.461	0.049	1.46×10^{-20}	0.492	0.912	1.064	0.291
T1w Corrected	0.461	0.049	1.46×10^{-20}	0.008	0.828	-0.194	0.230
multimodal Uncorrected	2.318	0.046	$<2.47\times10^{-324}$	0.283	0.824	-2.119	0.199
multimodal Corrected	2.318	0.046	$<2.47\times10^{-324}$	0.116	0.783	-2.146	0.172

The raw (unstandardized beta values) time point differences were corrected for age, sex, the interaction between age and sex, as well as the scanner site.
R^2m refers to marginal variance explained and R^2c refers to conditional variance explained.

Supplementary Table 4.a Associations between cross-section and longitudinal measures (Principal Components and BAGs)

	Beta	Std. Beta	SE	t	p
**dMRI corrected BAG**	-0.010	0.006	0.006	-1.497	0.027
**T1w corrected BAG**	0.028	0.005	0.005	5.511	3.94×10^{-8}
**Multimodal corrected BAG**	0.001	0.006	0.006	0.235	0.814
**dMRI uncorrected BAG**	-0.016	0.005	0.005	-2.855	0.004
**T1w uncorrected BAG**	0.028	0.005	0.005	5.475	4.81×10^{-8}
**Multimodal uncorrected BAG**	0.001	0.006	0.006	0.235	0.814
**dMRI PC**	-0.063	0.015	0.015	-4.075	4.75×10^{-5}
**T1w PC**	-0.001	0.018	0.018	-0.077	0.938
**Multimodal PC**	-0.054	0.017	0.017	-3.205	0.001

Std. Beta indicates the standardized β coefficients, SE the Standard Error. All brain age gap (BAG) associations are between the centercept of the BAG ($B\bar{A}G$), i.e. the cross-sectional BAG, and the annual rate of change of BAG a(ΔBAG) i.e., the longitudinal BAG. Similarly, all principal component (PC) associations were between the centercept of the PC ($\bar{PC}$), i.e. the cross-sectional PC, and the annual rate of change PC (ΔPC) i.e., the longitudinal PC. The associations were controlled for the inter-scan interval, age, sex, the age-sex interaction, and the scanner/acquisition site.

Supplementary Table 4.b Associations between cross-section and longitudinal measures (Principal Components and BAGs) controlling for inter-scan interval interaction

For completion, we also include a table for the effects when modelling the interaction effect with a cubic spline with k = 4 knots, using generalised additive models. F and p values in the last two columns on the right indicate the test statistics for the non-linear interaction effects. The left side of the table can be read as the table above.

	**Effect of the centercept**				**Interaction Term**	
	**Std.Beta**	**SE**	**t**	**p**	**F**	**p**
dMRI corrected BAG	-0.033	0.020	-1.629	0.103	2.890	0.002
T1w corrected BAG	0.105	0.019	5.463	5.14×10^{-8}	0.434	0.713
multimodal corrected BAG	0.007	0.019	0.383	0.701	1.024	0.310
dMRI uncorrected BAG	-0.059	0.020	-2.941	0.003	2.950	0.028
T1w uncorrected BAG	0.145	0.027	5.382	8.04×10^{-8}	2.363	0.089
multimodal uncorrected BAG	0.009	0.022	0.414	0.679	3.592	0.010
dMRI PC	-0.081	0.020	-4.097	4.33×10^{-5}	2.295	0.092
T1w PC	0.003	0.023	0.131	0.896	2.651	0.050
multimodal PC	-0.068	0.022	-3.171	0.002	5.436	0.020

Supplementary Table 5. Associations between cross-section and longitudinal measures (Principal Components and BAGs) accounting for the interaction of the inter-scan interval and $\bar{BAG}$

	Sex-ISI Interaction				Centercept			
	Std. Beta	SE	t	p	Std. Beta	SE	t	p
dMRI corrected BAG	-0.020	0.027	-0.744	0.457	0.019	0.069	0.277	0.781
T1w corrected BAG	0.009	0.023	0.385	0.700	0.084	0.060	1.396	0.163
multimodal corrected BAG	-0.016	0.026	-0.613	0.540	0.044	0.067	0.655	0.513
dMRI uncorrected BAG	-0.032	0.031	-1.040	0.298	0.021	0.078	0.271	0.786
T1w uncorrected BAG	0.004	0.025	0.163	0.871	0.137	0.067	2.056	0.040
multimodal uncorrected BAG	-0.027	0.026	-1.041	0.298	0.072	0.067	1.060	0.289
dMRI PC	-0.016	0.025	-0.632	0.527	-0.042	0.065	-0.634	0.526
T1w PC	0.006	0.026	0.227	0.821	-0.016	0.067	-0.240	0.811
multimodal PC	0.062	0.027	2.331	0.020	-0.221	0.069	-3.223	0.001

ISI indicates the Inter-Scan Interval, Std. Beta indicates the standardized β coefficients, SE the Standard Error. All brain age gap (BAG) associations are between the centercept of the BAG ($\bar{BAG}$), i.e. the cross-sectional BAG, and the annual rate of change of BAG a(ΔBAG) i.e., the longitudinal BAG. Similarly, all principal component (PC) associations were between the centercept of the PC ($\bar{PC}$), i.e. the cross-sectional PC, and the annual rate of change PC (ΔPC) i.e., the longitudinal PC. The associations were controlled for the inter-scan interval (and the interaction between the inter-scan interval and the respective cross-sectional measure), age, sex, the age-sex interaction, and the scanner/acquisition site.

Supplementary Table 6. Associations between Brain Age Gaps (longitudinal and cross-sectional) and Principal Components (longitudinal and cross-sectional)

Variable Pair	Std. Beta	SE	t	p
corrected $\bar{BAG}$_dMRI & ΔPC_dMRI	0.002	0.020	0.107	0.915
corrected $\bar{BAG}$_dMRI & $\bar{PC}$_dMRI	0.230	0.019	11.821	2.04×10^{-31}
corrected $\bar{BAG}$_T1w & ΔPC_T1w	0.077	0.022	3.549	3.94×10^{-4}
corrected $\bar{BAG}$_T1w & $\bar{PC}$_T1w	0.453	0.023	19.727	1.19×10^{-80}
corrected $\bar{BAG}$_multi & ΔPC_multi	0.003	0.020	0.170	0.865
corrected $\bar{BAG}$_multi & $\bar{PC}$_multi	0.311	0.021	14.740	3.15×10^{-47}
uncorrected $\bar{BAG}$_dMRI & ΔPC_dMRI	0.046	0.020	2.322	0.020
uncorrected $\bar{BAG}$_dMRI & $\bar{PC}$_dMRI	0.004	0.020	0.187	0.852
uncorrected $\bar{BAG}$_T1w & ΔPC_T1w	0.054	0.015	3.549	3.94×10^{-4}
uncorrected $\bar{BAG}$_T1w & $\bar{PC}$_T1w	0.317	0.016	19.727	1.19×10^{-80}
uncorrected $\bar{BAG}$_multi & ΔPC_multi	0.003	0.018	0.170	0.865
uncorrected $\bar{BAG}$_multi & $\bar{PC}$_multi	0.273	0.019	14.740	3.15×10^{-47}
corrected ΔBAG_dMRI & ΔPC_dMRI	0.004	0.020	0.224	0.823
corrected ΔBAG_dMRI & $\bar{PC}$_dMRI	0.011	0.020	0.571	0.568
corrected ΔBAG_T1w & ΔPC_T1w	0.471	0.019	25.082	4.39×10^{-124}
corrected ΔBAG_T1w & $\bar{PC}$_T1w	0.039	0.024	1.618	0.086
corrected ΔBAG_multi & ΔPC_multi	0.010	0.020	0.518	0.605
corrected ΔBAG_multi & $\bar{PC}$_multi	-0.024	0.021	-1.109	0.267
uncorrected ΔBAG_dMRI & ΔPC_dMRI	0.004	0.020	0.224	0.823
uncorrected ΔBAG_dMRI & $\bar{PC}$_dMRI	0.011	0.020	0.571	0.568
uncorrected ΔBAG_T1w & ΔPC_T1w	0.434	0.019	23.108	2.75×10^{-107}
uncorrected ΔBAG_T1w & $\bar{PC}$_T1w	0.035	0.023	1.503	0.133
uncorrected ΔBAG_multi & ΔPC_multi	0.010	0.020	0.518	0.605
uncorrected ΔBAG_multi & $\bar{PC}$_multi	-0.024	0.021	-1.109	0.267

The associations were controlled for the inter-scan interval, age, sex, the age-sex interaction, and the scanner/acquisition site.

Supplementary Table 7. Model performance comparison

Model	r2	MAE	RMSE	cor	CI95l	CI95u
LM T1 training	0.510	4.338	5.393	0.714	0.709	0.719
LM T1 TP1	0.472	4.530	5.356	0.687	0.669	0.707
LM T1 TP2	0.482	4.574	5.345	0.687	0.669	0.706
LM dMRI training	0.761	2.995	3.915	0.872	0.870	0.875
LM dMRI TP1	0.709	3.197	4.027	0.829	0.812	0.847
LM dMRI TP2	0.722	2.976	3.879	0.844	0.823	0.863
LM multi training	0.779	2.855	3.564	0.883	0.880	0.884
LM multi TP1	0.773	2.985	3.554	0.883	0.869	0.893
LM multi TP2	0.739	2.893	3.581	0.864	0.847	0.880
XGB T1 training	0.694	3.121	4.003	0.764	0.761	0.768
XGB T1 TP1	0.583	3.389	4.367	0.640	0.615	0.664
XGB T1 TP2	0.575	3.634	4.516	0.620	0.593	0.644
XGB dMRI training	0.704	2.975	3.837	0.776	0.771	0.781
XGB dMRI TP1	0.605	3.298	4.246	0.676	0.652	0.699
XGB dMRI TP2	0.582	3.449	4.369	0.670	0.648	0.691
XGB multi training	0.722	2.895	3.646	0.797	0.794	0.801
XGB multi TP1	0.597	3.172	4.192	0.773	0.746	0.797
XGB multi TP2	0.575	3.438	4.360	0.783	0.767	0.794
Lasso T1 training	0.470	4.156	5.345	0.687	0.683	0.690
Lasso T1 TP1	0.536	4.374	5.157	0.715	0.698	0.732
Lasso T1 TP2	0.616	3.745	4.878	0.746	0.730	0.760
Lasso dMRI training	0.695	2.943	3.726	0.780	0.776	0.784
Lasso dMRI TP1	0.659	3.279	4.133	0.752	0.735	0.770
Lasso dMRI TP2	0.693	2.820	3.723	0.780	0.767	0.791
Lasso multi training	0.790	2.840	3.465	0.895	0.892	0.899
Lasso multi TP1	0.676	3.198	3.992	0.794	0.782	0.801
Lasso multi TP2	0.631	3.746	4.686	0.794	0.791	0.798

Supplementary Table 8. White matter features by diffusion approaches

Diffusion Approach	Metrics
Bayesian Rotationally Invariant Approach (BRIA)[1]	intra-axonal axial diffusivity (DAX intra)
	extra-axonal radial diffusivity (DRAD extra)*
	microscopic fractional anisotropy (micro FA)
	extra-axonal axial diffusivity (DAX extra)
	intra-axonal water fraction (V intra)
	extra-axonal water fraction (V extra)
	cerebrospinal fluid fraction (vCSF)
	microscopical axial diffusivity (micro AX)
	microscopic radial diffusivity (micro RD)
	microscopical apparent diffusion coefficient (micro ADC)
Diffusion Kurtosis Imaging (DKI)[2, 3]	mean kurtosis (MK)
	radial kurtosis (RK)
	axial kurtosis (AK)
Diffusion Tensor Imaging (DTI)[4]	fractional anisotropy (FA)
	axial diffusivity (AD)
	mean diffusivity (MD)
	radial diffusivity (RD)
Spherical Mean Technique (SMT)[5]	fractional anisotropy (SMT FA)
	mean diffusivity (SMT md)
	transverse diffusion coefficient (SMT trans)
	longitudinal diffusion coefficient (SMT long)
Multi-compartment Spherical Mean Technique (SMTmc)[6]	extra-neurite microscopic mean diffusivity (SMTmc extra md)
	extra-neurite transverse microscopic diffusivity (SMTmc extra trans)
	mc SMTdiffusion coefficient (SMT mcd)
	intra-neurite volume fraction (SMTmc intra)
White Matter Tract Integrity (WMTI)[3]	axonal water fraction (AWF)
	radial extra-axonal diffusivity (radEAD)
	axial extra-axonal diffusivity (axEAD)*

*Note that Drad extra and axEAD were excluded from the analyses as a significant portion of the produced metrics did not pass our quality control procedure[7].

Supplementary Table 9. Within time point predictions from T1w data

**Variable**	**Value**
Predicted age difference	-3.7500234809095
Corrected predicted age difference	-6.0001114317298
Age difference	2.45381110190555
Brain age gap time point 1	9.61243510239738
Brain age gap time point 2	3.40860051958232
Corrected brain age gap time point 1	9.61243510265176
Corrected brain age gap time point 2	3.40860051970726
t-test p-value	0.00271397321264959
LMER p-value	0.0643570751142024
LMER beta	-3.86480438957065
Marginal means	Decreasing, non-sign.

Table 1 Statistical Measurements and Values

Supplementary Table 10. Between time point predictions from T1w data (uncorrected BAG)

Prediction	PredictedAge	ChronologicalAge	BAG
TP1 to TP1	71.82037	62.22277	9.612435
TP2 to TP2	68.07034	64.66812	3.408601
TP1 to TP2	92.35542	62.22277	30.147489
TP2 to TP1	76.35155	64.66812	11.689805

Table 2 Placeholder caption.

Supplementary Table 10. Between time point predictions from T1w data (corrected BAG)

Table 3 Placeholder caption for age predictions

Prediction	CorrectedPredictedAge	ChronologicalAge	CorrectedBAG
TP1 to TP1	71.82037	62.22277	9.612435
TP2 to TP2	68.07034	64.66812	3.408601
TP1 to TP2	47.34363	62.22277	-17.318117
TP2 to TP1	58.10051	64.66812	-4.107422

SUPPLEMENTARY FIGURES

Supplementary Figure 1. Scree Plots for the Principal Components Analyses

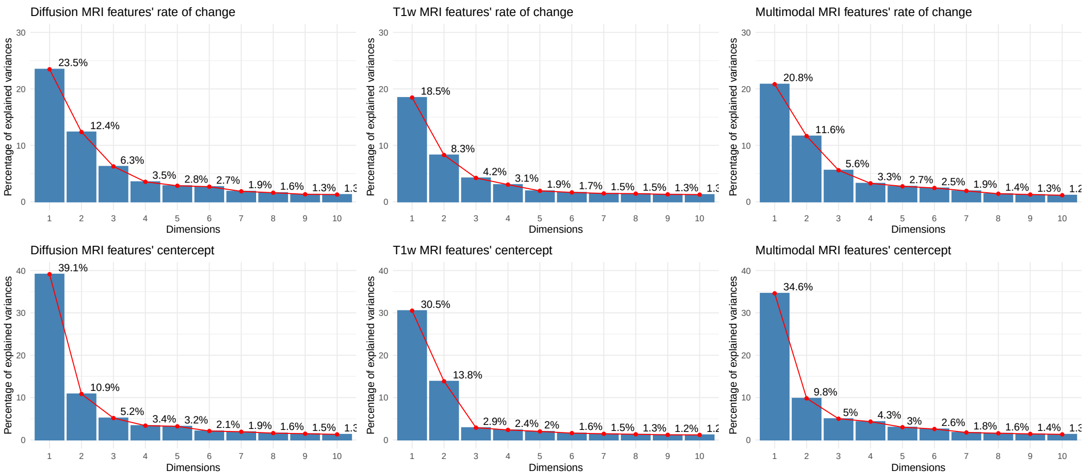

Supplementary Figure 2. Contour plots visualising the interaction effect of inter-scan interval and cross-sectional BAG on the annual rate of change of BAG

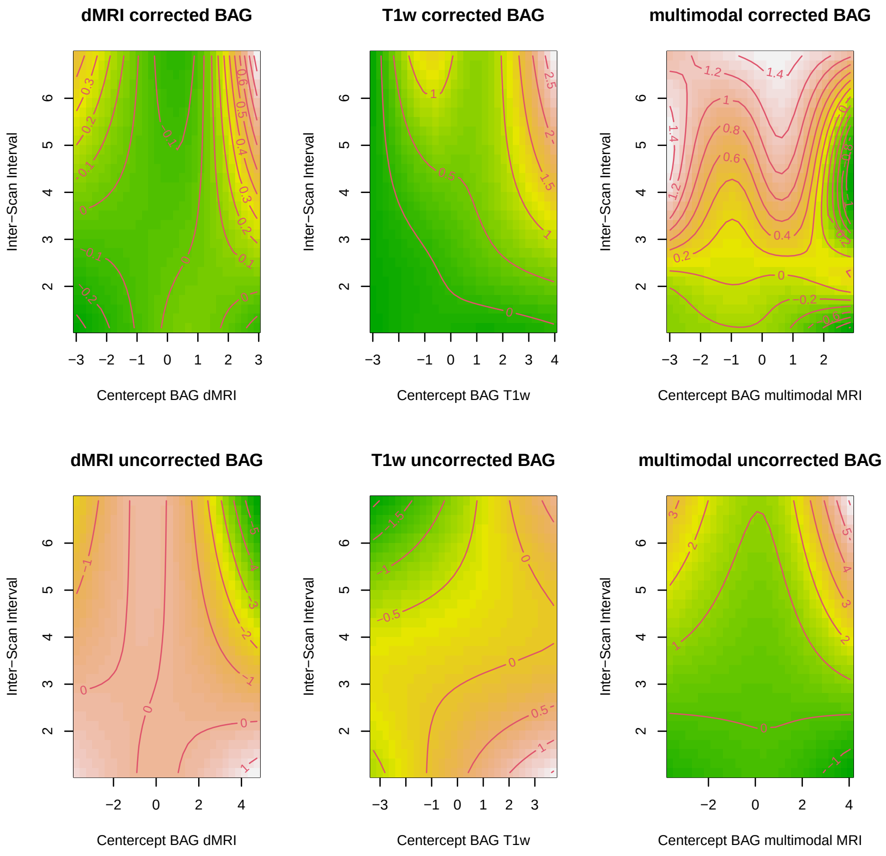

Supplementary Figure 3. Relative contribution of features to the first two principal components for each modality

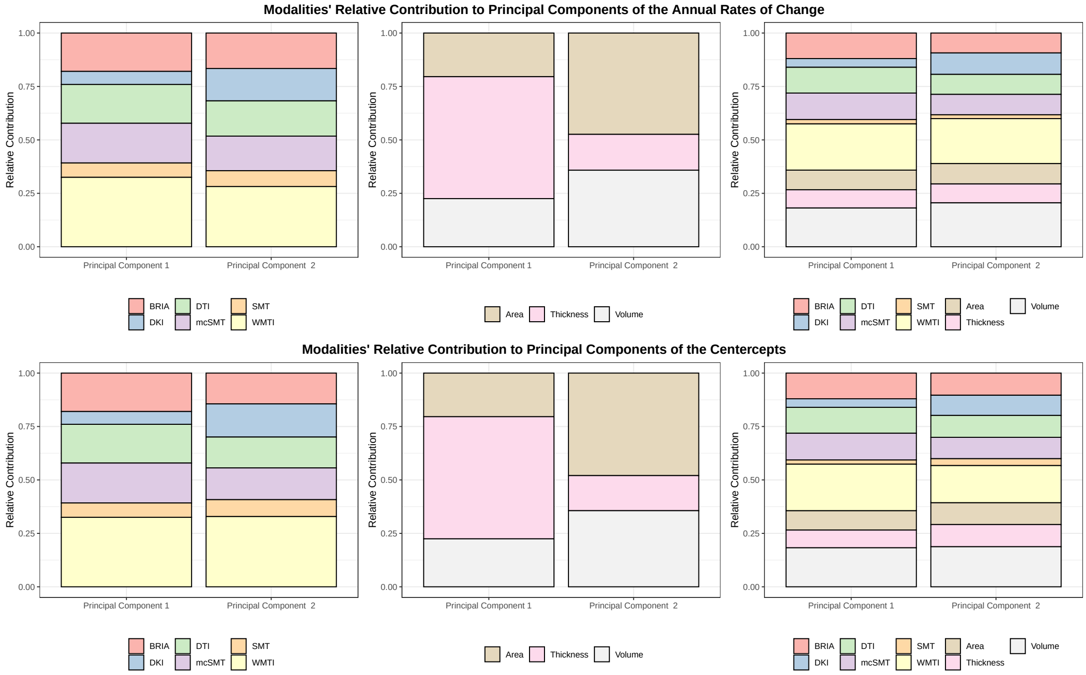

Panels from left to right: Diffusion MRI, T_1-weighted MRI, multimodal MRI. Diffusion features were derived using different approaches, including Diffusion Tensor Imaging (DTI) [4], Diffusion Kurtosis Imaging (DKI) [2], White Matter Tract Integrity (WMTI) [3], Spherical Mean Technique (SMT) [5], and multi-compartment Spherical Mean Technique (mcSMT)[6], and the Bayesian Rotational Invariant Approach (BRIA)[1].

Supplementary Figure 4. Associations between PCs, BAG, annual BAG and PC change, and pheno- and geno-types of health

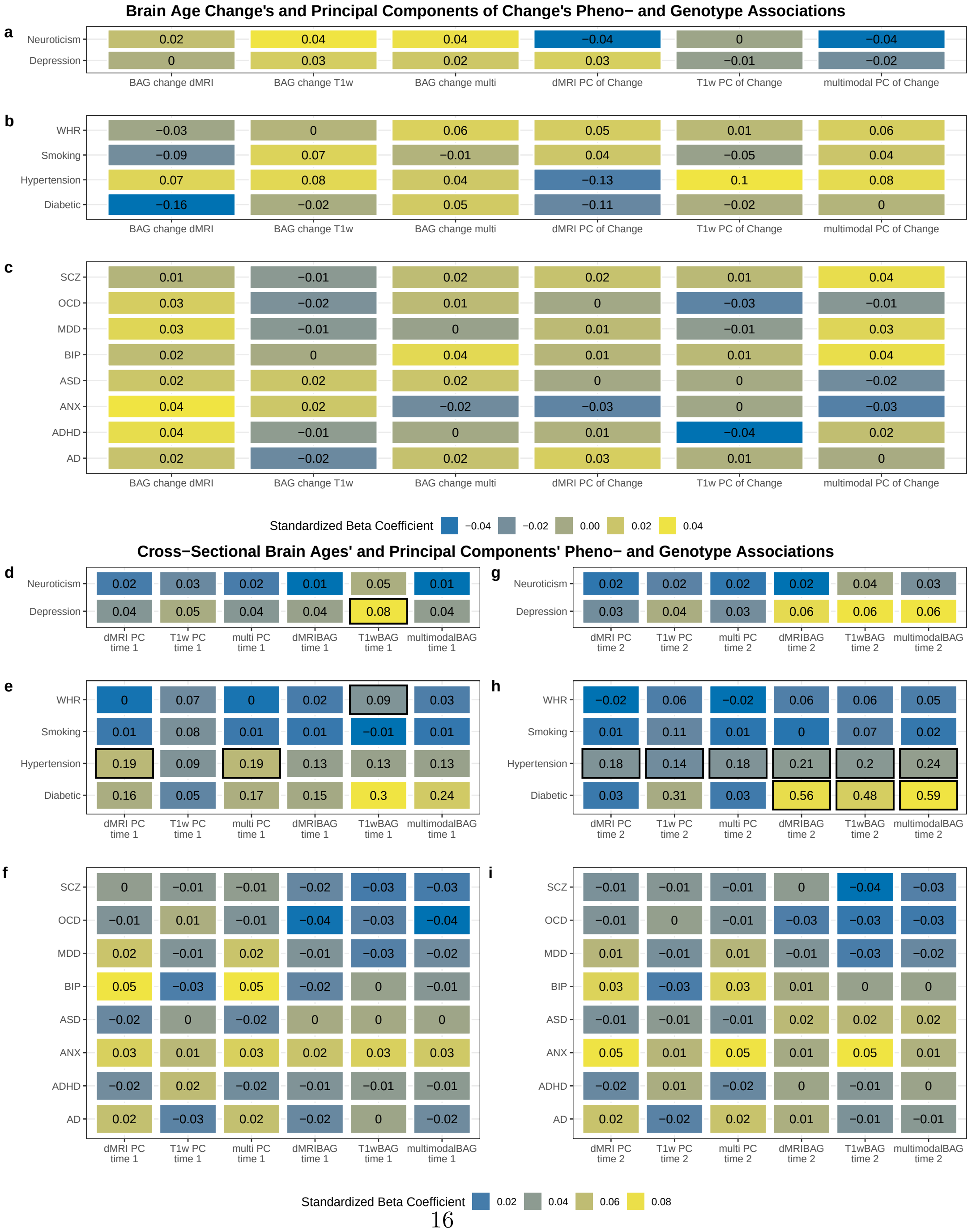

a) Associations between the annual change in multimodal principal components (PCs) and brain age gaps (BAGs) with neuroticism and depression. b) Associations between the annual change in multimodal PCs and BAGs with cardiometabolic risk factors. WHR = waist to hip ratio, Smoking (yes/no), Hypertension (yes/no), Diabetes (yes/no). c) Associations between the annual change in multimodal PCs and BAGs with polygenic risk scores of psychiatric disorders and Alzheimer's disease. SCZ = schiziphrenia, OCD = obsessive compulsive disorder, MDD = major depressive disorder, BP = bipolar disorder, ASD = autism spectrum disorder, ANX = anxiety disorder, ADHD = attention deficit hyperactivity disorder, AD = Alzheimer's Disease. d-e) Associations as presented in a-c) but with cross-sectional measures of PCs and BAGs.

Note: Framed boxes indicate an FDR-corrected $p < 0.05$. Colours indicate the direction of the associations (blue = negative, yellow = positive). The effect sizes (standardised beta coefficients) are indicated in each box.

Supplementary Figure 5. Time point differences in features by T1-weighted metrics and diffusion approaches

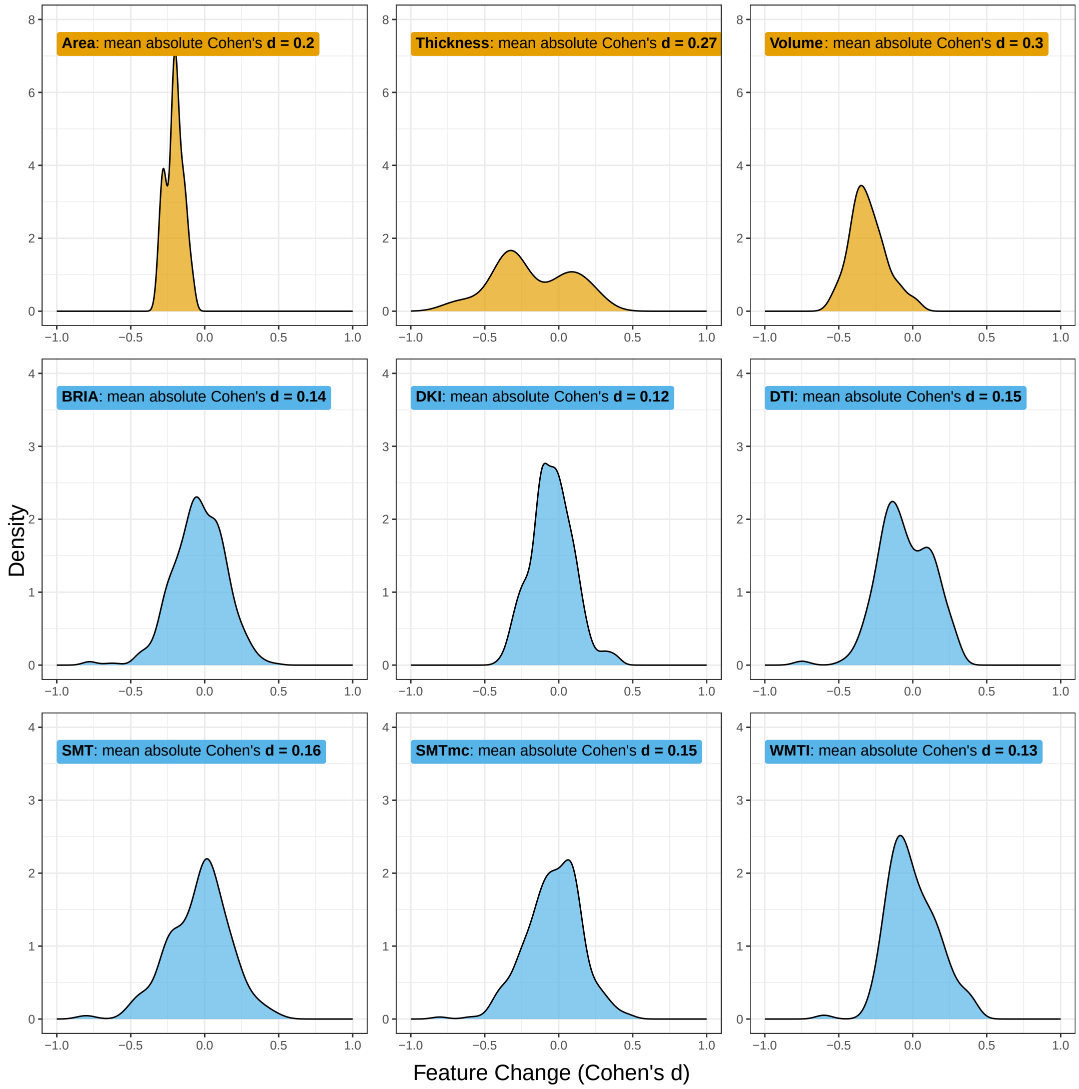

The top row shows the distribution of time point differences in $T_1 - weighted$ MRI derived metrics. The bottom two rows show the distribution of time point differences in all metrics entailed in each of the selected dMRI approaches.

Supplementary Figure 6. Time point differences in features by processing approach

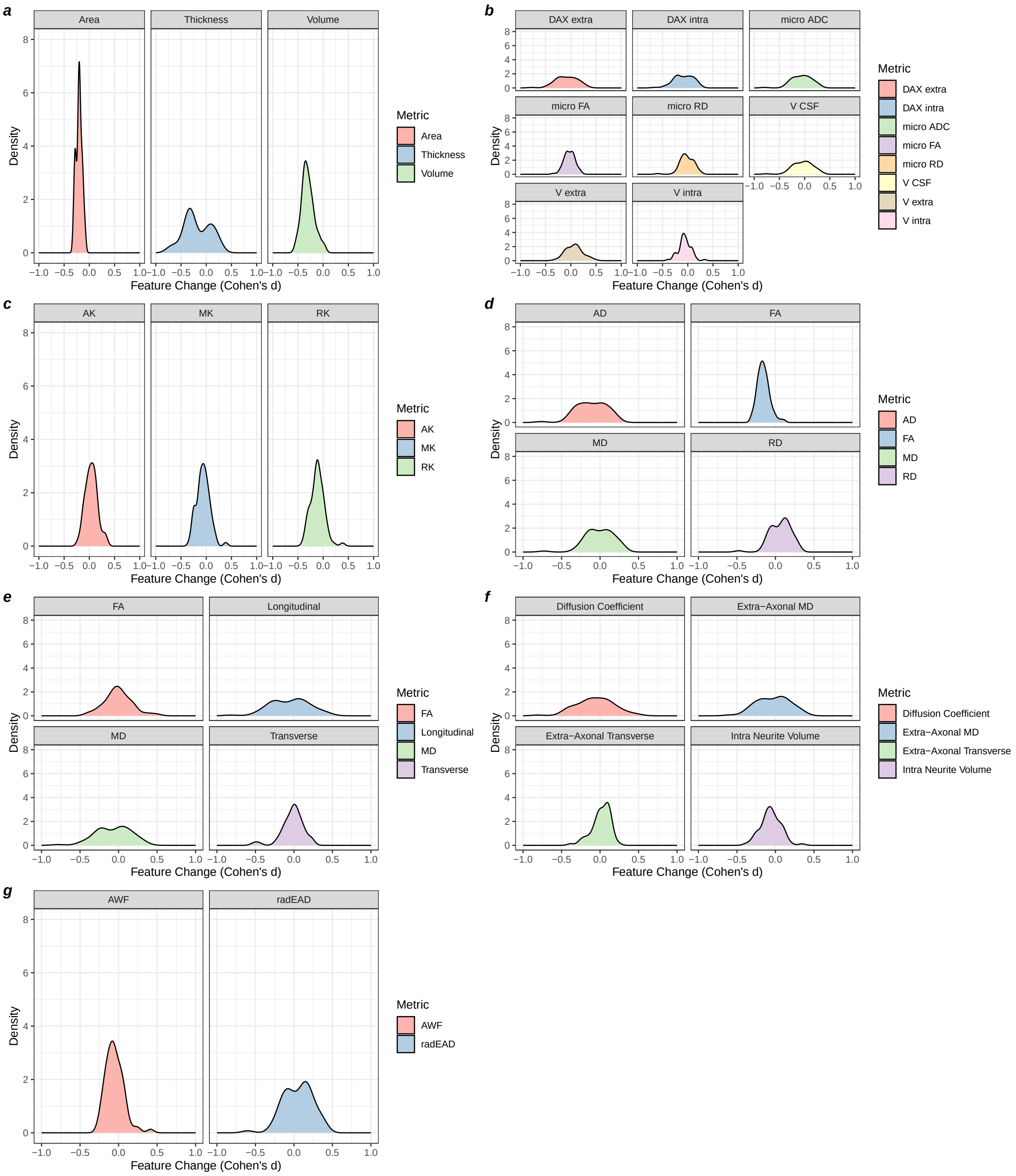

The figure presents the distribution of regional changes in different metrics. a) T_1w MRI derived metrics, b) metrics from the Bayesian Rotationally Invariant Approach,

c) metrics from Diffusion Kurtosis Imaging, d) metrics from Diffusion Tensor Imaging, e) metrics derived using the Spherical Mean Technique, f) metrics derived using the multi-compartment variant of Spherical Mean Technique, g) metrics from the White Matter Tract Integrity approach.

Among dMRI metrics, fractional anisotropy ($\bar{d} = -0.16$), radial kurtosis ($\bar{d} = -0.11$), axial diffusivity ($\bar{d} = -0.11$), extra- ($\bar{d} = -0.09$) and intra-axonal diffusivity ($\bar{d} = -0.09$) showed the greatest changes between regions (Suppl. Fig., 6). Among the T_1-weighted metrics, regional volume had the largest change between baseline and follow-up ($\bar{d} = -0.30$), followed by cortical surface area ($\bar{d} = -0.20$), and thickness ($\bar{d} = -0.18$) (Suppl. Fig. 6), both corresponding to previously mapped age-trends [8–10].

Supplementary Figure 7. Relative contributions of feature types to principal components by modality

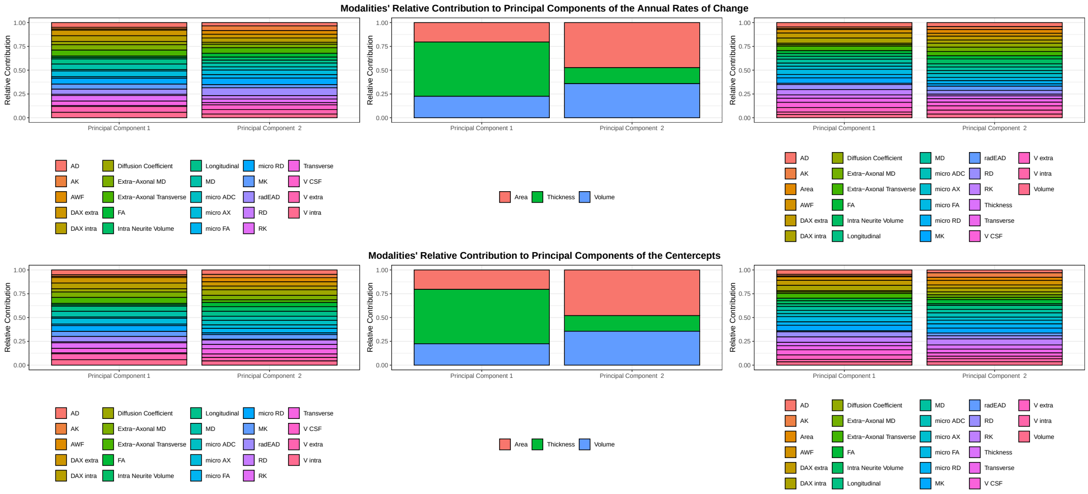

SUPPLEMENTARY NOTES

Supplementary Note 1. Analysis protocol

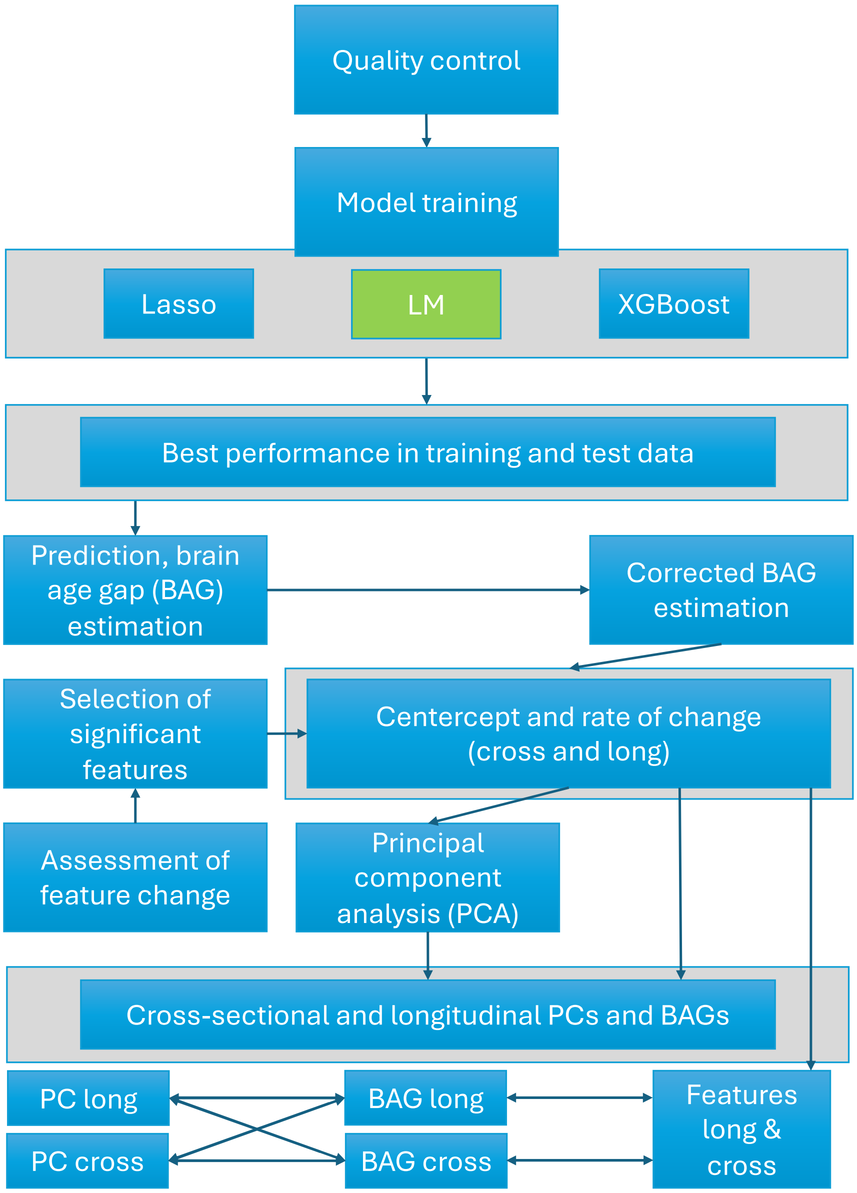

1. Data processing and extraction of region-wise metrics/features, QC. Resulting data: a large cross-sectional training data set and a longitudinal data set with two time point serving as test data. We differentiate data in diffusion MRI derived metrics

only, T1-weighted MRI derived metrics only, and their combination, labelled as multimodal MRI data.

2. Training of brain age models from these features, using XG Boost, Lasso, and linear models.
3. Assessment of the trained models based on predictions in training and test samples. Selection of the best-performing models.
4. Predictions in test data were obtained. Additionally, the predictions were corrected by the slope of linear models associating age and brain age. This results in uncorrected and corrected brain age estimates.
5. Brain age gaps (BAG) were estimated from these estimates by subtracting chronological age from the brain age estimates.
6. For the analyses, we first estimate the time point differences and correlations between time point specific BAGs.
7. We then assessed the effects of feature changes using t-tests and quantify the effects using Cohen's d. We average the obtained d values by modality (dMRI, T1-weighted, multimodal MRI) and then by metric (e.g., volume, surface area, fractional anisotropy).
8. For the significantly changing features the centercept and the annual rate of change in the features were estimated.
9. The resulting centercept and rate of change feature-level metrics were used to estimate principal components of which we used the first component each: one component reflecting the centercept, one reflecting the annual rate of change in features.
10. Similarly, we estimated the centercept (as predictor) and annual rate of change of BAGs which were associated with each other.
11. Additionally, the centercept (as predictor) and annual rate of change of PCs were associated with each other.
12. As a control, the same two analyses above were executed using the inter scan interval to be interacted with the centercept.
13. Then, we associated the centercepts of BAG with the annual rate of change in the PCs, both within modality (e.g., BAG_{dMRI} and ΔBAG_{dMRI}) and between modalities (e.g., BAG_{dMRI} and ΔBAG_{T1w}).
14. As a final step, we associated the centercepts and annual rates of changes in BAGs and PCs with the centercepts and annual rates of changes of the features which significantly changed over time.

REFERENCES

[1] Reisert, M., Kellner, E., Dhital, B., Hennig, J. & Kiselev, V. G. Disentangling micro from mesostructure by diffusion MRI: a Bayesian approach. *Neuroimage* **147**, 964–975 (2017).

[2] Jensen, J. H., Helpern, J. A., Ramani, A., Lu, H. & Kaczynski, K. Diffusional kurtosis imaging: the quantification of non-gaussian water diffusion by means of magnetic resonance imaging. *Magnetic Resonance in Medicine: An Official Journal of the International Society for Magnetic Resonance in Medicine* **53**, 1432–1440 (2005).

[3] Fieremans, E., Jensen, J. H. & Helpern, J. A. White matter characterization with diffusional kurtosis imaging. *Neuroimage* **58**, 177–188 (2011).

[4] Basser, P. J., Mattiello, J. & LeBihan, D. Mr diffusion tensor spectroscopy and imaging. *Biophysical journal* **66**, 259–267 (1994).

[5] Kaden, E., Kruggel, F. & Alexander, D. C. Quantitative mapping of the per-axon diffusion coefficients in brain white matter. *Magnetic resonance in medicine* **75**, 1752–1763 (2016).

[6] Kaden, E., Kelm, N. D., Carson, R. P., Does, M. D. & Alexander, D. C. Multi-compartment microscopic diffusion imaging. *NeuroImage* **139**, 346–359 (2016).

[7] Maximov, I. I. *et al.* Fast qualitY conTrol meThod foR derIved diffUsion Metrics (YTTRIUM) in big data analysis: UK Biobank 18,608 example. *HBM* **42**, 3141–3155 (2021).

[8] Korbmacher, M. *et al.* Brain asymmetries from mid-to late life and hemispheric brain age. *Nature Communications* **15**, 956 (2024).

[9] Korbmacher, M. *et al.* Brain-wide associations between white matter and age highlight the role of fornix microstructure in brain ageing. *HBM* **44** (2023).

[10] Korbmacher, M. *et al.* Distinct longitudinal brain white matter microstructure changes and associated polygenic risk of common psychiatric disorders and alzheimer's disease in the uk biobank. *Biological Psychiatry Global Open Science* 100323 (2024).
